# Spatial lipidomics reveals biased phospholipid remodeling in acute *Pseudomonas* lung infection

**DOI:** 10.1016/j.isci.2023.107700

**Published:** 2023-08-21

**Authors:** Alison J. Scott, Shane R. Ellis, Casey E. Hofstaedter, Ron M.A. Heeren, Robert K. Ernst

**Affiliations:** 1 Department of Microbial Pathogenesis, University of Maryland School of Dentistry, Baltimore, MD 21201, USA; 2 Maastricht MultiModal Molecular Imaging (M4i) Institute, Maastricht University, 6200 MD Maastricht, the Netherlands; 3 Molecular Horizons and School of Chemistry and Molecular Bioscience, University of Wollongong, Wollongong, NSW 2522, Australia

**Keywords:** Biological sciences, Microbiology, Lipidomics

## Abstract

*Pseudomonas aeruginosa* (*Pa*) is a pathogen causing chronic pulmonary infections in patients with cystic fibrosis (CF). Manipulation of lipids is an important feature of *Pa* infection and on a tissue-level scale is poorly understood. Using a mouse model of acute *Pa* pulmonary infection, we explored the whole-lung phospholipid response using mass spectrometry imaging (MSI) and spatial lipidomics. Using a histology-driven analysis, we isolated airways and parenchyma from both mock- and *Pa*-infected lungs and used systems biology tools to identify enriched metabolic pathways from the differential phospholipid identities. Infection was associated with a set of 26 ions, with 11 unique to parenchyma and 6 unique to airways. Acyl remodeling was differentially enriched in infected parenchyma as the predominant biological function. These functions correlated with markers of polymorphonuclear (PMN) cell influx, a defining feature of the lung response to *Pa* infection, implicating enzymes active in phospholipid remodeling.

## Highlights

• Divergent lipid response to *Pseudomonas aeruginosa* (*Pa*) infection in mouse lung
• Analysis showed different lipid response in infected airways versus parenchyma
• Pathway analysis revealed acyl remodeling function bias in infected parenchyma
• Neutrophils overlapped with acyl remodeling activities, pointing to therapeutic target

## Graphical abstract

**Figure:**
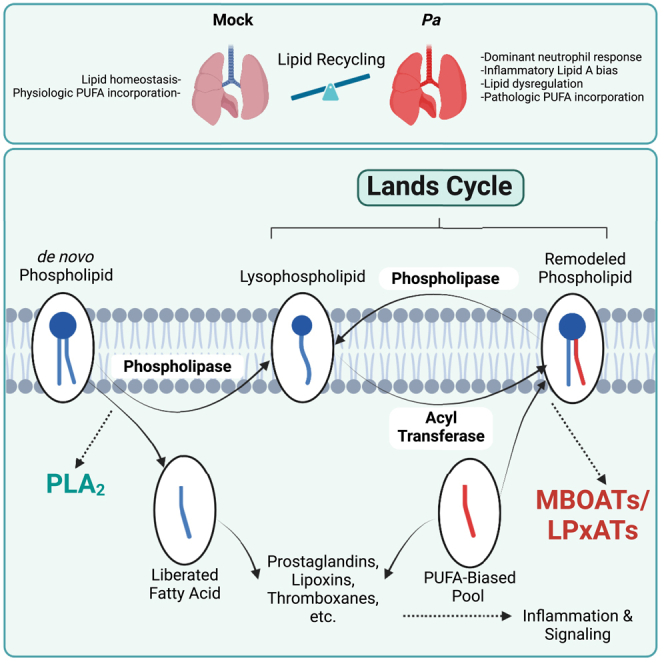

## Introduction

Mass spectrometry imaging (MSI) is a rapidly expanding method of -omic level profiling that preserves spatial distribution and is now commonly used to profile host-pathogen interactions. We used a combination of MSI modalities to resolve the differential spatial lipidomic^1^^,^^2^^,^^3^ profile in mouse lungs in response to acute infection by *Pseudomonas aeruginosa* (*Pa*). Infection by *Pa* is a lifelong complication in patients with cystic fibrosis (CF)^4^^,^^5^^,^^6^ and with the emergence of multi- and extensively drug-resistant *Pa* strains,^7^ identifying new mechanisms to control the infection are of critical need. We previously demonstrated the ability of MSI to describe a lethal pathogenic mechanism in a mouse model of Tularemia using differential lipid maps from mouse spleen, establishing that MSI can illuminate lethal pathogenic mechanisms of bacterial infection from lipids alone.^8^ Further, in that model, the anion-forming phospholipids (PLs) were sufficient to inform pathogenic mechanisms.^8^ In this work, we focused on the lipid ions detected in negative ion mode by MALDI-TOF mass spectrometry. We analyzed the infected lung samples using MALDI-TOF at 50 μm spatial resolution to illustrate the spatial elements of the infection. In parallel, we used a data-dependent acquisition (DDA)-MSI method to generate lipidome-per-pixel images to structurally identify the lipids within the infected lungs.^3^ Together, these data formed the basis for a systems biology type analysis^9^ identifying dysregulation of the lipid profile as a central component of the mouse pulmonary response to *Pa* infection.

Lipids are the major constituents of biological membranes and are influenced by intracellular and extracellular conditions. A growing number of studies show a role for the dysregulation of host lipids in CF airway inflammation, lipid metabolism, and disease. In CF, patients have increased production of prostaglandin E2 metabolites and thromboxane B2 and its metabolites at all ages, suggesting a role for PL-derived metabolites in CF airway pathogenesis.^10^ Additionally, the *Pa* virulence factor, cystic fibrosis transmembrane conductance regulator (CFTR) inhibitory factor (Cif), promotes sustained airway inflammation by reducing host pro-resolving lipid mediators.^11^ Cif hydrolyzes epithelial-derived 14,15-epoxyeicosatrienoic acid, disrupting transcellular production of the pro-resolving lipid 15-epi lipoxin A_4_ (15-epi LXA_4_) by neutrophils. Clinical data from CF patients revealed that Cif abundance correlated with increased inflammation, decreased 15-epi LXA_4_, and reduced pulmonary function. Finally, a growing body of research suggests a significant role for the recycling of polyunsaturated fatty acid (PUFA)-containing phospholipids, linking specific phospholipids to ferroptosis and inflammation.^12^

The Lands cycle^13^^,^^14^ is a route for membrane phospholipid recycling (Graphical Abstract). The fatty acids of phospholipids are enzymatically removed (by a phospholipase), and the liberated fatty acid can have many fates including conversion to prostaglandins, thromboxanes, lipoxygenases, and others involved in lipid inflammation and, eventually (given the right conditions), pro-resolving mediators.^15^^,^^16^^,^^17^ When a fatty acid is removed from a phospholipid, what remains in a lysophospholipid that on its own has diverse downstream roles. However, to maintain the membrane phospholipid pool, the lysophospholipid can be re-acylated by an acyltransferase family enzyme—reconstituting an intact phospholipid. While four different processes are involved in the Lands cycle (based on various dependencies for acyl-coenzyme A), the two main functions are de-acylation and re-acylation. This process diversifies the cellular phospholipid pool, the result of which can impact membrane-dependent functions. Due to an historical absence of tools to spatially resolve this process *in situ*, little is known about the role of the Lands cycle and dysregulation thereof in the context of a CF lung infection. The Lands cycle enzymes are highly correlated with neutrophil function,^15^^,^^18^ and dysregulation of this pathway is consistent with the type of immune response observed in this model. To further extend the translational aspect of these results, ibuprofen is effective in relieving symptoms in young patients with CF, with the mechanism of ibuprofen reducing production of prostaglandins downstream of the Lands cycle.^19^ These studies highlight a critical role for lipid manipulation during *Pa* infection and represent druggable targets.

CF lung disease is characterized by CFTR dysfunction, decreased mucociliary clearance, and chronic inflammation. Bacterial infections cause much of the damage seen in CF patients’ airways. However, a substantial portion of the lung damage is caused by the activation of the host innate immune system that accompanies infection, specifically neutrophil activation.^20^ Few animal models of CF airway infection recapitulate this neutrophil predominance and dysfunction due to differences in CFTR expression between humans and mice. Neutrophils primarily drive this chronic inflammation through the release of pro-inflammatory mediators, myeloperoxidase, and neutrophil elastase (NE).^21^ In the absence of pathogenic bacteria, airway mucus plugging and hypoxia promote interleukin-8 (IL-8) and leukotriene B_4_ (LTB_4_) release from respiratory epithelial cells, increasing neutrophil migration to the lung. In the lung, neutrophils exacerbate the inflammatory milieu, while further stimulating epithelial cells to secrete more IL-8 and LTB_4_, recruiting more neutrophils to the lung.^22^^,^^23^ Once individuals with CF are colonized with *Pa*, this pro-inflammatory cycle worsens, leading to permanent lung damage and respiratory decline. Although the CF lung has increased neutrophil levels at the time of initial infection, CFTR-deficient neutrophils are less effective at bacterial clearance through numerous mechanisms.^24^ Additionally, CF neutrophils are more likely to undergo NETosis, the formation of neutrophil extracellular traps (NETs). NETosis is a mechanism of pro-inflammatory cell death where chromosomal DNA and NE are extruded from the neutrophil.^24^ While NETosis may be an effective way of infection control in other disease states, in CF it is ineffective and further worsens chronic inflammation and lung damage. An improved depth of cellular and molecular characterization of this host-pathogen interaction is necessary to better understand the mechanisms involved, and ultimately, to develop an improved bactericidal response from the host by targeting host processes.

In this study, we used an acute *Pa* infection model in wild-type mice to demonstrate the spatially dependent differential lipid responses in the lungs and identify new host-directed therapeutic targets.

## Results

### Differential ion fingerprint in infected lungs

**Figure 1:**
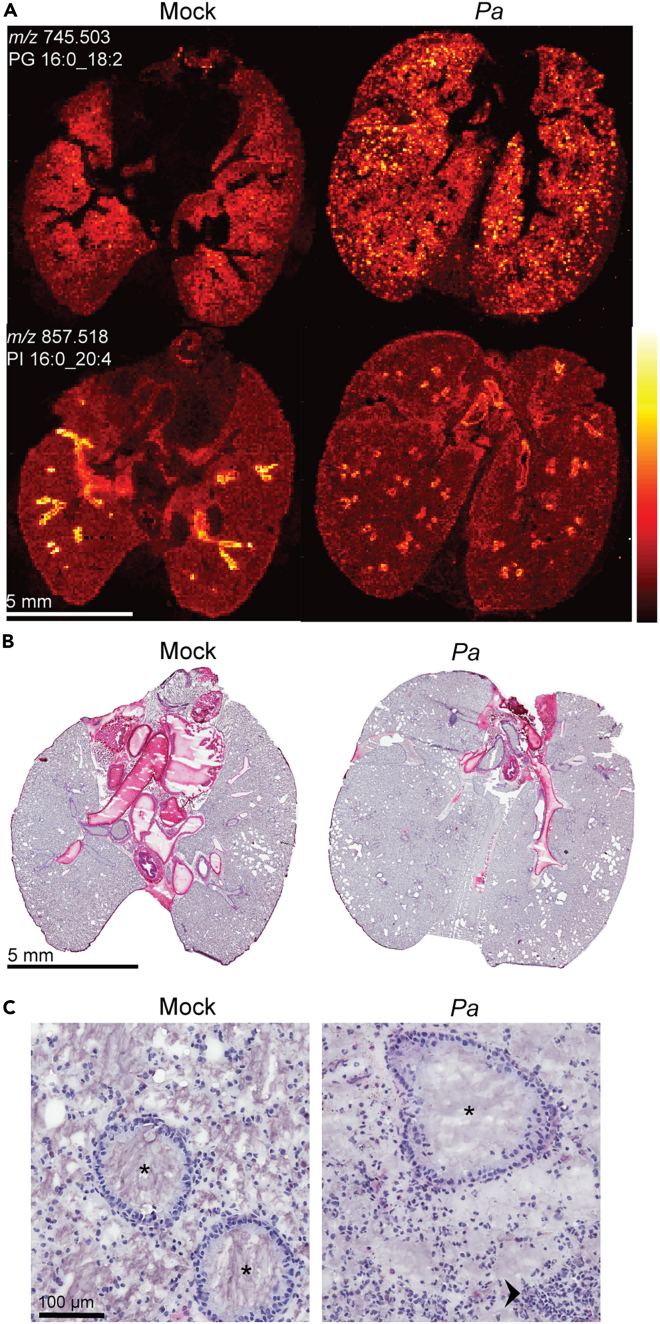
Representative images and reference histology from tissue segmentation (A) Projection of polyunsaturated fatty acid (PUFA)-containing phospholipids, ions *m/z* 745.503 and 857.518 in both mock- and *Pa*-infected lungs from DDA-MSI experiment. Ion identities as given ([M-H]- for both). Representative of hundreds of lipid ions in dataset. Normalized, total ion current (TIC), scale bar 0–100% rel. intensity. Negative ion mode, MALDI-ELITE, 80 μm. (B) H&E reference stain from post-MSI tissues analyzed in (A). (C) Representative airway (AW, asterisk) and parenchyma (PCM, unmarked) regions manually segmented for histology-driven differential lipid detection. Arrowhead: dense polymorphonuclear (PMN) cellular infiltrate typical in the PCM of *Pa*-infected lungs.

**Figure 2:**
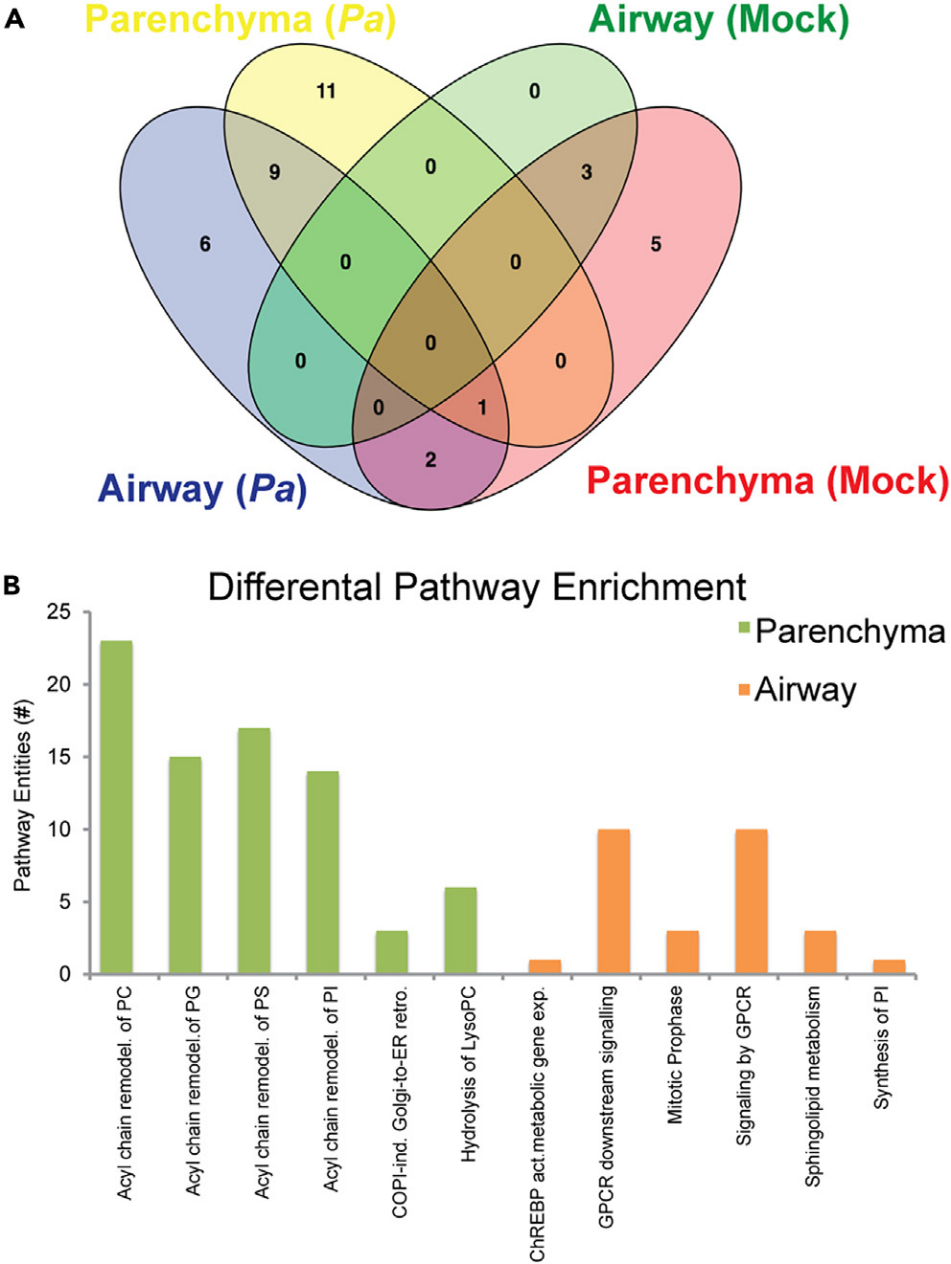
Regional analysis of differential lipids and identification of enriched pathway functions in *Pa*-infected PCM (A) Venn diagram showing total unique ions describing mock- or *Pa*-infected segments. (B) Differential and significant pathway enrichment in *Pa*-infected PCM compared to AW segment. Statistical analysis based on MALDI-TOF triplicate dataset, negative ion mode, 50 μm spatial resolution.

We used histological features to instruct segmentation of 2 regions of interest (ROIs) from each of the datasets: airway (AW) and parenchyma (PCM) from *Pa*-infected or mock-infected mouse lungs (Figure S1). Slides were stripped of matrix following MSI data capture, stained with H&E, optically scanned at 20x, and the tissue architecture was used to determine AW and PCM areas (Figure 1). The resulting ROIs contained 638 pixels (+/− 41) for AW and 1794 pixels (+/− 101) in the PCM. Average mass spectra for each ROI were exported, recalibrated, averaged (by group, mock- or *Pa*-infected), and normalized, and ion intensities were compared between groups (Figure 2) resulting in a pattern of 26 ions enriched in the *Pa*-infected AW and PCM components, non-overlapping with any mock-infected region. In the AW ROI, 6 ions were associated with *Pa* infection and no ions uniquely associated with mock infection or overlapping both conditions. The PCM ROI showed 11 ions enriched in the *Pa*-infection condition compared to 5 associated only with mock infection. A total of 9 ions were associated with *Pa*-infected AW and PCM. These data demonstrated a pattern of enrichment of specific ions with *Pa*-infected AW and PCM.

### Phospholipid fingerprint of infected lungs

Using the ion fingerprints associated with each functional ROI and experimental group, we assigned putative identities using a combination of: i) on-tissue fragmentation using TOF/TOF, ii) accurate mass and ion trap MS/MS fragmentation obtained using DDA-imaging, and iii) previous curation in bacterial infection in mouse models. When ion abundance thresholds could not be met for satisfactory MS/MS identification, putative identities were inferred from bulk extracts using the most abundant isomer that could be identified using the criteria defined previously. Phospholipids were the predominant class identified (Table 1) with nearly half (8 of 17) confirmed as polyunsaturated. Representative data from two ions from the triplicate MALDI-TOF dataset are given in Figure S2. While phosphatidylethanolamine (PE) and phosphatidylglycerol (PG) are the predominant classes of phospholipids found in the *Pa* membrane,^25^ these lipids are not exclusive to the bacteria and were included in the analysis. However, the sum composition 34:1 (carbons:unsaturations) is the most abundant composition usage used by *Pa*, *in vitro*, and it is possible that their enrichment in the *Pa*-infected PCM lung ROI is directly attributable to the bacteria.^25^ Extensive use of PUFA, notably arachidonic acid (C20:4), an omega-6 fatty acid, was found in the *Pa*-infected PCM, along with evidence for preferential incorporation of oleic (C18:1) and linoleic (C18:2, also omega-6) acids. Extensive incorporation of PUFAs is not found in lipid extracts of *Pa*.

**Table 1:** Lipid identities comprising differential fingerprint

Ion (*m/z*)	Association	Assignment Used	HMDB ID
647.5^#^	*Pa* PCM & AW	PA 16:0/16:0	HMDB0000674
673.5^#^	*Pa* PCM & AW	PA 16:0_18:1 PA 18:1_16:0	HMDB0007859 HMDB0114924
717.5 719.5^#∗^	*Pa* AW *Pa* AW	PG 16:1_16:1 PG 16:0_16:1 PG 16:1_16:0	HMDB0010586 HMDB0010571 HMDB0010585
721.5^#^	Mock AW	PG 16:0_16:0	HMDB0010570
723.5^#^	*Pa* AW	PA 18:0_20:4 PA 20:4_18:0	HMDB0114884 HMDB0115149
743.5^#^	*Pa* PCM & AW	PG 16:1_18:2 PG 18:2_16:1	HMDB0010590 HMDB0010646
773.5^#^	*Pa* AW	LBPA 18:1/18:1	N/A
774.5^#^	Mock AW & PCM	PE P-18:0/22:6	HMDB0011394
778.5^#^	Mock PCM	PE 19:1_20:4 PE 20:4_19:1	N/A N/A
780.8	*Pa* PCM & AW	PE 19:0_20:4 PE 20:4_19:0	N/A N/A
786.5^#^	*Pa* PCM	PS 18:0_18:2 PS 18:2_18:0	HMDB0012380 HMDB0012400
788.5^#^	Mock AW & PCM	PS 18:0_18:1 PS 18:1_18:0	HMDB0010163 HMDB0012389
795.5	*Pa* AW	PG 16:0_22:5 PG 22:5_16:0	N/A N/A
798.5	*Pa* PCM & AW	PG 18:0_20:3 PG 20:3_18:0	HMDB0010609 N/A
812.5^#^	*Pa* PCM & AW	PS 18:0_20:3 PS 20:3_18:0	HMDB0012382 HMDB0012422
833.5^#^	*Pa* PCM & AW	PI 16:0_18:2 PI 18:2_16:0	HMDB0009784 HMDB0009846
838.6^#^	*Pa* PCM & AW	PS 18:0_22:4 PS 22:4_18:0	HMDB0112382 HMDB0112793
857.5^#∗^	Mock AW	PI 16:0_20:4 PI 20:4_16:0	HMDB000#### HMDB000####
885.5^#∗^	Mock AW & PCM	PI 18:0_20:4 PI 20:4_18:0	HMDB0009815 HMDB0009894

Ions implicated in differential assignments, associated with *Pa* infection (AW and PCM). Assignments: differential ions identified in triplicate MALDI-TOF dataset. Ions referenced to DDA-imaging dataset^#^ and/or confirmed with MS/MS fragment data^∗^.

### Lands cycle implicated in infected lungs

**Figure 3:**
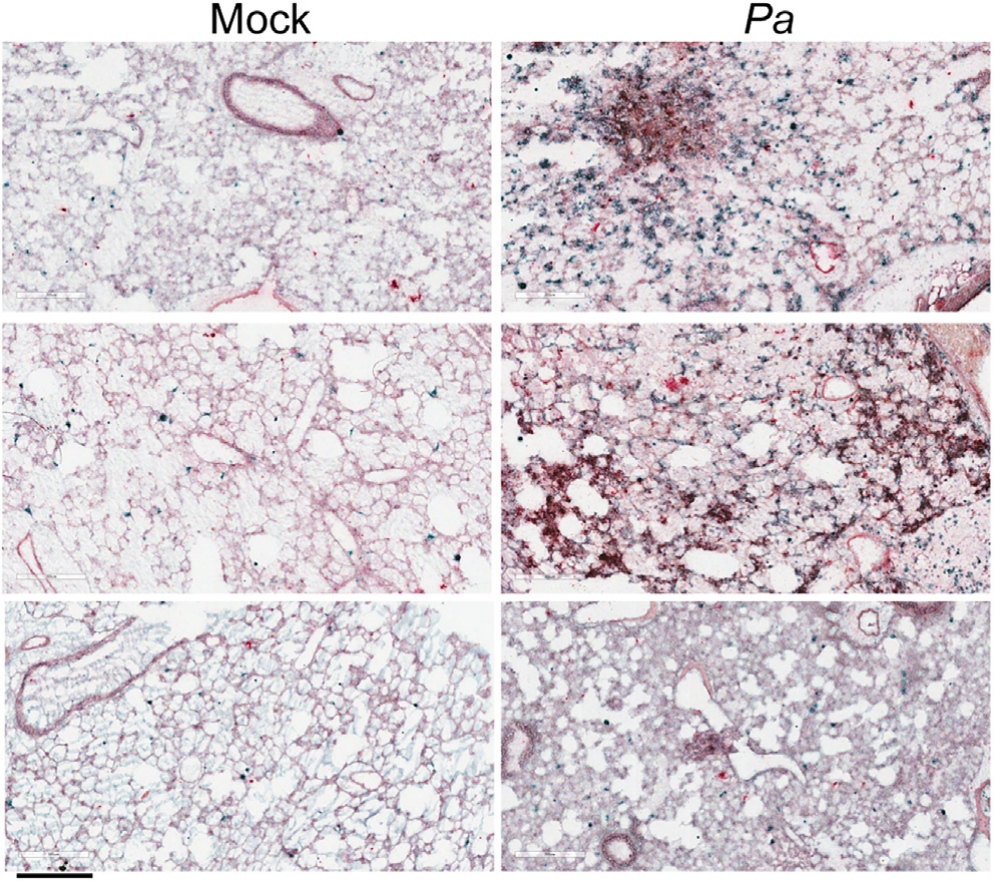
Dense infiltration of infected lungs by neutrophils co-localized with *Pa* bacteria Triplicate lungs from mock- (left) and *Pa*-infected (right) serial sections from MALDI-TOF dataset stained with anti-Ly6G [clone 1A8] (emerald green) and anti-*Pa* (red) with light Mayer’s hematoxylin counterstain. Capture areas representative of overall tissue section and shown from an 8x field view in a predominantly PCM area. Scale bar: 300 μm.

Lipid fingerprints (Figure 2) were assigned metabolite identities using the human metabolite database identifiers,^26^ with any ambiguities parsed as described in the STAR Methods (Table 1). Not all differential ions could be confidently assigned and were omitted. A metabolite-protein orthologous interaction network was constructed using the human KEGG knowledgebase for each of the ROI types (AW and PCM; Figures S3 and S4). For *Pa*-infected AW, we observed two interaction nodes: phosphatidylserine (PS) and phosphatidic acid (PA) indicating potential protein interactors (Figures S4B and S4C; Table S1). In the *Pa*-infected PCM, both the PS and PA nodes from the AW network were found and a third, PE node was found indicating potential protein interactors (Figure S4A; Table S1). We assembled the lists of protein interactors suggested by the network analysis into generalized enzymatic function with any known ligand specificity (Table S1). Next, we queried the lists of protein interactors generated from the metabolite-protein interaction network for enrichment of generalized pathways. Two functions predominated: lysolipid acyltransferases and *sn*2-position phospholipases (Table S2). These two classes of enzymes implicate active involvement of the Lands cycle and are an active component of neutrophil responses; neutrophils are the primary cellular infiltrate in *Pa*-infected lungs. By immunohistochemistry (IHC), we observed a robust neutrophilic influx in the PCM of *Pa*-infected lungs compared to mock-infected controls (Figure 3). Further, in PCM regions of dense cellular influx, neutrophils are co-localized with abundant *Pa* antigen. Notably, both *Pa* organisms are present as dense microcolonies in these regions along with *Pa* antigen-positive areas lacking extensive intact organisms indicating incomplete neutrophil-mediated killing at 48 h. These results highlight a key role of lipid immunometabolism in the neutrophil-mediated response to *Pa* infection.

## Discussion

Chronic pulmonary infections by *Pa* are a major cause of morbidity in patients with CF and chronic obstructive pulmonary disease. There is a gap in knowledge surrounding the fundamental host response to *Pa* infection in the lung from the perspective of lipids and metabolites. Here, we used an acute *Pa* infection in wild-type mouse lungs to determine whether histologically segmented lipidomic data could identify novel patterns of lipid metabolism to advance into studies of chronic *Pa* infection in CF models.

In this study, we focused on the role of phospholipids detected from tissue in the negative ion mode only. Our previous data from infected spleens demonstrated that negative ion mode phospholipids were sufficient to illustrate innate immune inflammation mechanisms. Though, given the important role in the lung of phospholipids that preferentially ionize in positive ion mode (ex: phosphatidylcholine, PC) as a major surfactant component, and the increasing links between lysoPCs and host response, future studies may benefit from including both positively and negatively ionized lipids. An important caveat of the DDA-MSI method is that precursor ion selection and the subsequent fragment scans are iterative across the entire tissue section. Therefore, the final ion identities are not necessarily sensitive to the location in which they were detected. It is possible that this contributes to some inconsistency in ion identification assigned in the TOF dataset as AW, but the identity used could have stemmed from an isobaric or isomeric precursor in the PCM, and vice versa. As a mitigating factor, the bulk of the spectra (based on area) in the DDA-MSI run would be in the PCM segment meaning the majority of identities would stem from the major region of interest. Finally, lacking stereospecific and double-bond positional isomer resolution in these experiments, we chose to include both possible stereoidentities in the metabolic network analysis, though many of the arrangements are improbable based on commonly observed stereospecificities and this will have an impact on the proposed enzyme involvement. We limited double-bond isomers to 9Z in cases where multiple positions were present in the metabolite library for monounsaturated acyl chains. Including multiple identities for a single ion interpretation could bias toward acyl remodeling by overrepresenting acyl possibilities, a possible limitation to this approach. This limitation is somewhat mitigated by the lack of acyl remodeling activities implicated in the AW segments, where the same metabolite identity and network analysis strategy was used. If the multiple assignments for a single ion biased the pathway analysis, then the acyl remodeling biases should be present in both AW and PCM, but it was a significantly enriched and differential feature of the PCM lipid profile, only.

The lung tissues evaluated here have a complex early immune response and the concomitant cellular and molecular changes of the host response are largely responsible for the differential lipid profiles during infection. However, *Pa* organisms are also present and replicating in abundance (evidenced by IHC results) and the total lipid response reflects some amount of bacterial lipids. The predominant acyl arrangement in lipid extracts from clinical and laboratory-adapted *Pa* strains is 34:1 with a high prevalence observed as the 16:0_18:1 molecular lipid species. The 34:1 composition is found on PE and PG head groups in lipid extracts from *Pa* with other minor head groups involved at far lower abundances.^25^ Notably, the 34:1 sum composition is generally absent from the differential lipid profiles of *Pa*-infected AW and PCM, lending strong support to the interpretation that the bulk of the differential lipid response is due to the immune response rather than replicating *Pa* organisms. Ultrahigh resolution MSI^27^ could address these issues in future studies, as practical and commonplace spatial resolution moves closer the cellular size of a single bacterium. Finally, an important next step will be to translate these results from acute infection to a model system that better reflects the features of the CF lung, for example the βENaC mouse model^28^ using mucoid clinical isolates of *Pa* to recapitulate important aspects of *Pa*-infected human lungs from patients with CF.

Here, we demonstrate a dysregulated lipid response to pulmonary infection with *Pa* in an acute infection model. The differential lipid profiles are spatially informed and demonstrate the differences between the lipid response to infection in the AW compared to the PCM. A pathway analysis of the enriched functions implicated by the differential lipid profiles supports a strong role for acyl remodeling as a central characteristic of the lung immune response to *Pa* infection. These results strongly highlight the need to move away from bulk extractions to characterize -omic level changes in histologically and functionally complex organs. Finally, enzymes of the Lands cycle are supported as potential targets for investigation of new host-directed therapies for *Pa* infection.

### Limitations of the study

Several limitations are present in this work. An acute pulmonary infection model does not accurately reflect all of the complexities of a chronic infection. These results only address an acute response and are the starting place for future work in chronic pulmonary infection models. Further, we could not separate the bacterial lipids from host lipids in this study and bacterial lipid changes could contribute, at least in part, to the overall differential lipid fingerprint. The neutrophil response was not correlated or directly overlaid with specific lipids, rather, the regional neutrophil responses (ex: parenchyma) were compared to the differential parenchymal lipid identities and this could limit the linkage to neutrophil-based mechanisms. In this study, we used an orthologous lipid:protein interaction network based on known human interactions and any mouse-specific activities would be underrepresented. While we identified acyl compositions in this work, finer details such as double-bond isomers and stereospecificities were not assigned. For this reason, we included both plausible *sn*-configurations while seeding the network analysis and that could contribute to the determination of acyl remodeling function. Finally, these studies were all carried out with a laboratory-adapted strain of *Pa* and in wild-type mice. Future work should focus on *Pa* clinical isolates and mouse strains that mimic the unique metabolic environment and immune responses of the CF lung.

## STAR★Methods

### Key resources table

**Table:** 

REAGENT or RESOURCE	SOURCE	IDENTIFIER
**Antibodies**		

Rat anti-Ly6G [1A8]	Abcam	ab210204
Rabbit anti-*Pa*	Abcam	ab68538

**Bacterial and virus strains**		

*Pseudomonas aeruginosa* PAK-1	Stephen Lory	N/A

**Chemicals, peptides, and recombinant proteins**		

Levamisole HCl	Abcam	ab141217
Lysogeny Broth (LB), Miller	Difco	244620
Magnesium Chloride, hexahydrate	Millipore Sigma	CAS 7791-18-6
Porcine Gelatin, ∼300 g bloom, Type A	Millipore Sigma	CAS 9000-70-8
Norharmane (NRM) matrix	Millipore Sigma	CAS 244-63-3
Mayer’s Hematoxylin	Abcam	ab220365

**Critical commercial assays**		

DoubleStain IHC Kit: R&Rt on human/mouse tissue (Green/HRP & Red/AP)	Abcam	ab183285

**Experimental models: Organisms/strains**		

Mouse, C57BL/6J, female adult	Jackson Labs	000664

**Software and algorithms**		

MSiReader v1.02	NC State Univ.	N/A

**Other**		

Indium tin oxide slides	Delta Technologies	5GS115

### Resource availability

#### Lead contact

Further information and requests for resources should be directed to the lead contact, Alison Scott (aliscott@umaryland.edu).

#### Materials availability

This study did not generate new unique reagents.

### Experimental model and study participant details

#### Preparation of bacteria

*Pseudomonas aeruginosa* strain PAK-1, a wildtype laboratory adapted strain originating from a burn wound isolate, was cultured from three plated colonies in 5mL lysogeny broth (LB) supplemented with 1 mM MgCl_2_ with orbital shaking (180 RPM) overnight (16-18 hours). PAK was obtained from Dr. Steven Lory, Harvard University. Bacteria were sub-cultured at a dilution of 1:200 using the same culture conditions for 3 hours, pellets were collected by centrifugation at 1000xg for 5 mins. Bacterial pellets were resuspended in sterile phosphate buffered saline (PBS, Gibco) and diluted to OD_600_ = 1 (approximately 10^8^ colony forming units, CFU, per mL) and a pellet was prepared as above from this bacterial slurry to deliver 10^8^ CFU in 10 μL of PBS.

#### Infection model

Wildtype C57BL/6 female mice (6-8 weeks old, 4 per group, Jackson Laboratories) were anesthetized with inhaled isoflurane and inoculated with 10 μL of the bacterial slurry (or sterile PBS for the mock-infected group) dropwise and alternating between nares. Mice were euthanized by carbon dioxide narcosis and lungs were prepared for MSI by inflation with 2% gelatin solution in water, float-frozen on liquid nitrogen, and stored at -80°C as previously described.^2^^,^^29^ All rodent work was performed under an approved IACUC protocol.

### Method details

#### MSI and DDA-imaging

Inflated lungs were sectioned at 13 μm and thaw-mounted onto indium-tin oxide (ITO) coated glass slides, sections were desiccated for 20 mins., and stored under nitrogen at -80°C prior to use. Slides were prepared for MSI by coating with norharmane (NRM, 7 mg/mL in 2:1, v:v, chloroform:methanol) matrix^30^^,^^31^ using an HTX Technologies TM Sprayer (Charlotte, NC) with the following cycle settings: 30°C, 10 passes, 0.1 mL/min, 1200 mm/min, 2.5 mm spacing, 10 psi N_2_, CC pattern, 0 s dry time, at 40 mm nozzle height. Coated slides were dried with a gentle nitrogen stream prior to imaging. Three biological replicates (arbitrarily selected) were evaluated by MALDI-TOF at 50 μm pixel size in negative ion mode on a Bruker RapiFlex (Bremen, DE) (Figure S1). On-tissue MS/MS fragmentations were performed on a Bruker RapiFlex. The fourth biological replicates from each group were evaluated by DDA-imaging in negative ion mode at 80 μm pixel size as previously described^3^ on a bespoke imaging instrument composed of a Spectroglyph dual MALDI/ESI ion source (Spectroglyph LLC, WA, USA) coupled to an Orbitrap Elite mass spectrometer (Thermo Fisher Scientific GmbH, Bremen, Germany). Mass resolution for MSI (MS^4^) data was 240,000 @ *m/z* 400. In addition to MSI data DDA-imaging data also provided high mass accuracy measurements and MS/MS spectra to aid lipid identification.

#### Immunostaining

Serial sections to the MSI slides were immunostained using two color visible dual staining. From storage at -80°C, sections were immersed in ice cold acetone for 10 mins with at least 3 up/down agitations. Fixed slides were dried completely in a vacuum desiccator for 10 mins. Sections were encircled with a hydrophobic pen (PAP Pen, Electron Microscopy Sciences). Sections were blocked with 5% normal goat serum in PBS-T (0.05% Tween 20 in PBS) for 15 mins. in a humidified chamber at ambient temperature. Endogenous peroxidases and phosphatases were blocked with 3% H_2_O_2_ (Fisher Scientific) containing 4 uM levamisole (Abcam) for 10 minutes in a humidified chamber at ambient temperature. Slides were rinsed twice in two brief changes of distilled water then washed thrice in PBS-T (2 mins. each). Primary antibodies were diluted 1:500 in 1% normal goat serum in PBS-T. *Pseudomonas* was detected with a polyclonal rabbit anti-*Pseudomonas* antibody and neutrophils with a monoclonal rat anti-Ly6G [1A8]. Primary antibodies were incubated overnight in a humidified chamber at 4°C and washed thrice in PBS-T as above. Dual color visible anti-rabbit (AP, red) and anti-rat (HRP, emerald) chromogen polymers were used to develop the images (Abcam anti-rat/anti-rabbit polymer kit) and performed exactly per manufacturer instructions. Diluted Mayer’s Hematoxylin (1:10 in water) was used to counterstain (30 second incubation) followed by rinsing in a reservoir with gently running tap water. Counterstain was blued for 10 seconds in PBS followed by rinsing in distilled water for 1 minute and including at least one change of water. Post-staining and counterstaining, slides were dehydrated [20 seconds each: 1x 85% ethanol, 1x 95% ethanol, 3x 100 ethanol, 1x xylenes] and coverslips mounted with Permount. Images were captured on an Aperio system and exported in ScanScope software (Leica).

### Quantification and statistical analysis

#### Data analysis

MALDI-TOF datasets were imported into MSIReader^32^^,^^33^ (v1.02) to annotate functional regions (airway, AW, or parenchyma, PCM) using a histology guided approach to generate regions of similar overall pixel numbers (300-500 total pixels for AW, 800-1100 pixels for PCM). Averaged spectra for each region type from each of the biological replicates were exported to mMass^34^ (v5.5.0) for detailed comparison and further evaluation. Spectra were recalibrated to known on-tissue ions in mMass. An average spectrum was calculated for mock- and *Pa*-infected PCM and AW regions. The averaged, normalized (total ion current, TIC) spectra were peak picked using the default mMass settings and compared to give a differential list of ions (mock versus *Pa*) for both regions using the following settings: fold change > 2 using peak intensity and minimum threshold intensity >10%. Peaks were putatively identified using ALEX123 lipid database^35^ seeded with negative ions and limited to the [M-H]^-^, as NRM results in nearly universal -H^-^ ions in negative mode. Fragmentation analysis supplemented the identify of ions with sufficient abundance to perform MS/MS directly from serial tissue sections. Finally, accurate mass measurements and MS/MS acquired using the Orbitrap Elite system and DDA data were used to confirm the identities of detected lipids. Identified lipids were transformed into metabolite identities using the human orthologs in the human metabolite database (HMDB). Whenever possible, if known, the acyl constituents were given (with the shorter acyl chain used at the *sn1* position for consistency) and all unsaturations of monounsaturated chains were assigned as n-9 because double-bond isomers were not resolved in these data. Polyunsaturated lipids were assigned based on previously observed assignments in Lipid Maps and evidence from literature reports whenever possible. Differential metabolite lists were assigned an association value (-2 associated with mock infection, +2 associated with *Pa* infection) and evaluated for protein-metabolite interactions from orthologous human interactions based on the KEGG knowledgebase^36^ using Omicsnet.^37^ Protein interacting partners implicated in the network analysis were seeded into Reactome^38^^,^^39^ by region type to identify functionally enriched pathways. Significantly enriched pathways (*p*<0.05 and false discovery rate, FDR<0.05) were evaluated by region type (workflow given in Figure S3).

## Data Availability

Data reported in this paper will be shared by the lead contact upon request. This paper does not report original code. Any additional information necessary to reanalyze the reported data is available from the lead contact upon request.
