# Supplementary material for: Spatial lipidomics reveals biased phospholipid remodeling in acute Pseudomonas lung infection
Source: iScience. 2023 Aug 21;26(9):107700. doi: 10.1016/j.isci.2023.107700 (PMC10480615; doi:10.1016/j.isci.2023.107700)
Supplement: Document S1. Figures S1–S4 and Tables S1 and S2 [file mmc1.pdf]

iScience, Volume *26*

Supplemental information

Spatial lipidomics reveals biased phospholipid remodeling in acute *Pseudomonas* lung infection

Alison J. Scott, Shane R. Ellis, Casey E. Hofstaedter, Ron M.A. Heeren, and Robert K. Ernst

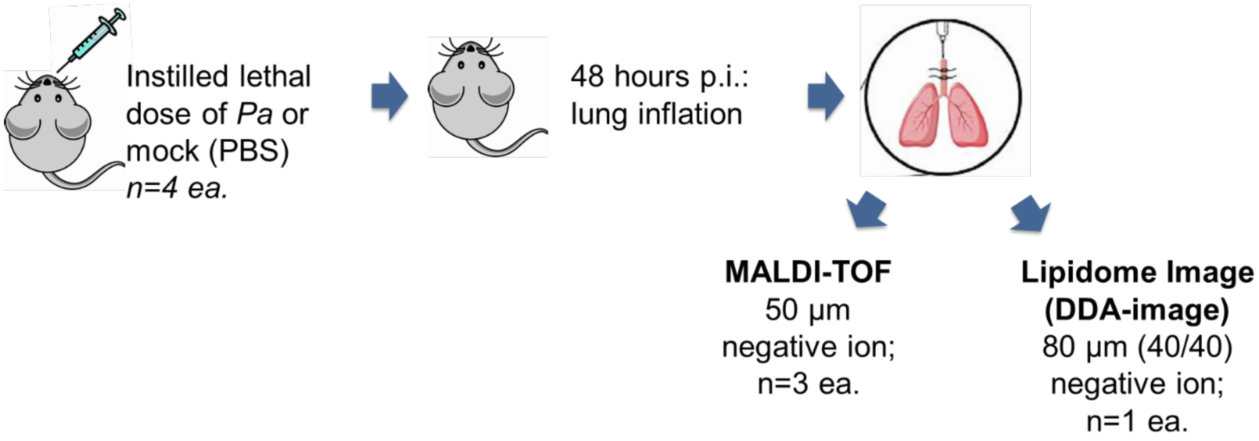

Figure S1: Experimental setup for acute *Pa* infection in wildtype mouse lung. Related Figures 1-3. General scheme of infection model and subsequent data collections. Note: mice were alive and showing moderate clinical signs of infection at 48 hours post-infection.

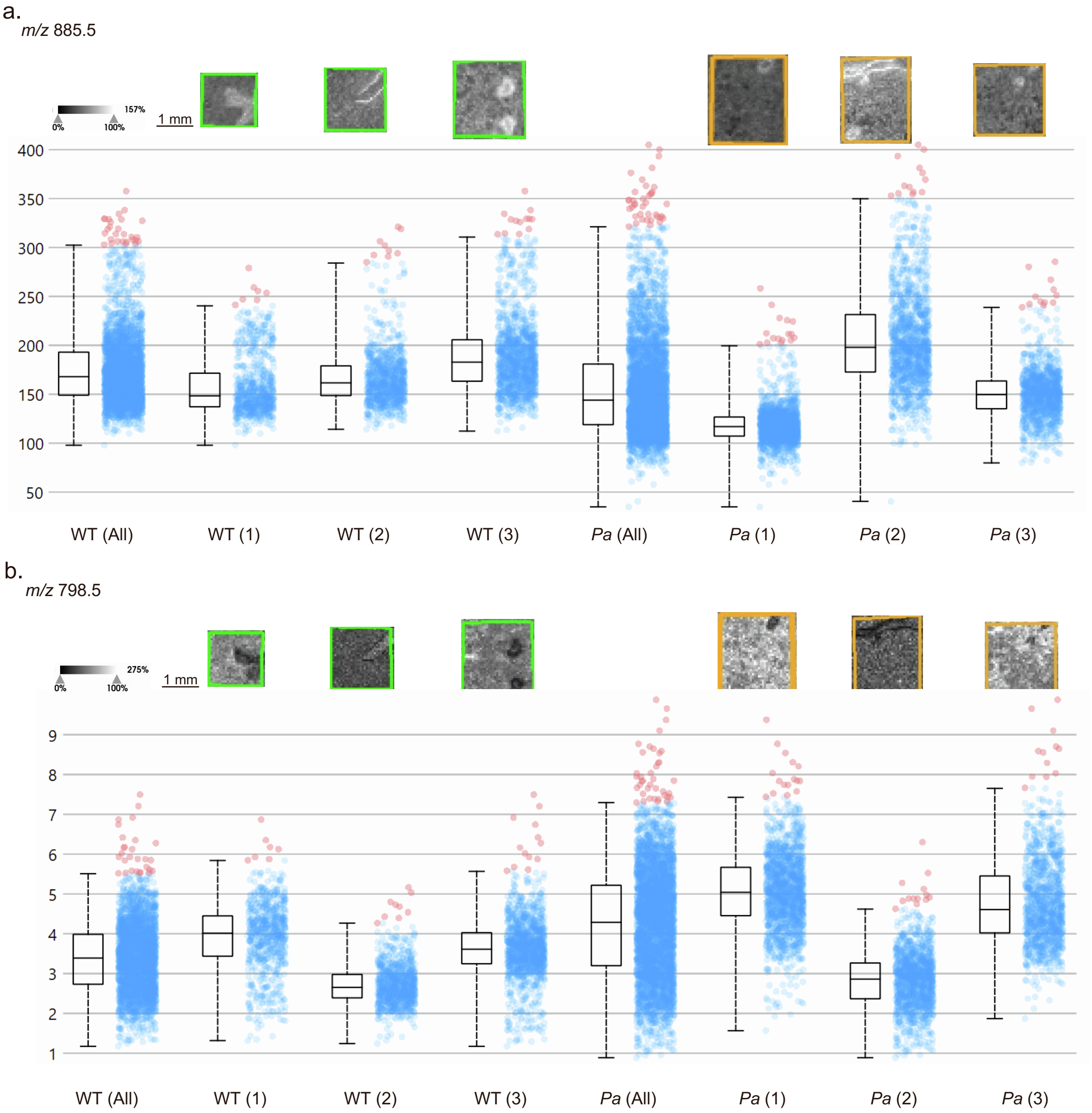

Figure S2: Snapshot of replicates from triplicate MALDI-TOF set. Related to Figure 1. Ions as given for a., b. showing lipid data from a region of the distal lung containing parenchyma and at least one airway feature. Similar total pixel areas as analyzed in differential analysis. Ion intensity distributions for each area showing triplicates together (All) and separately. Normalized (TIC).

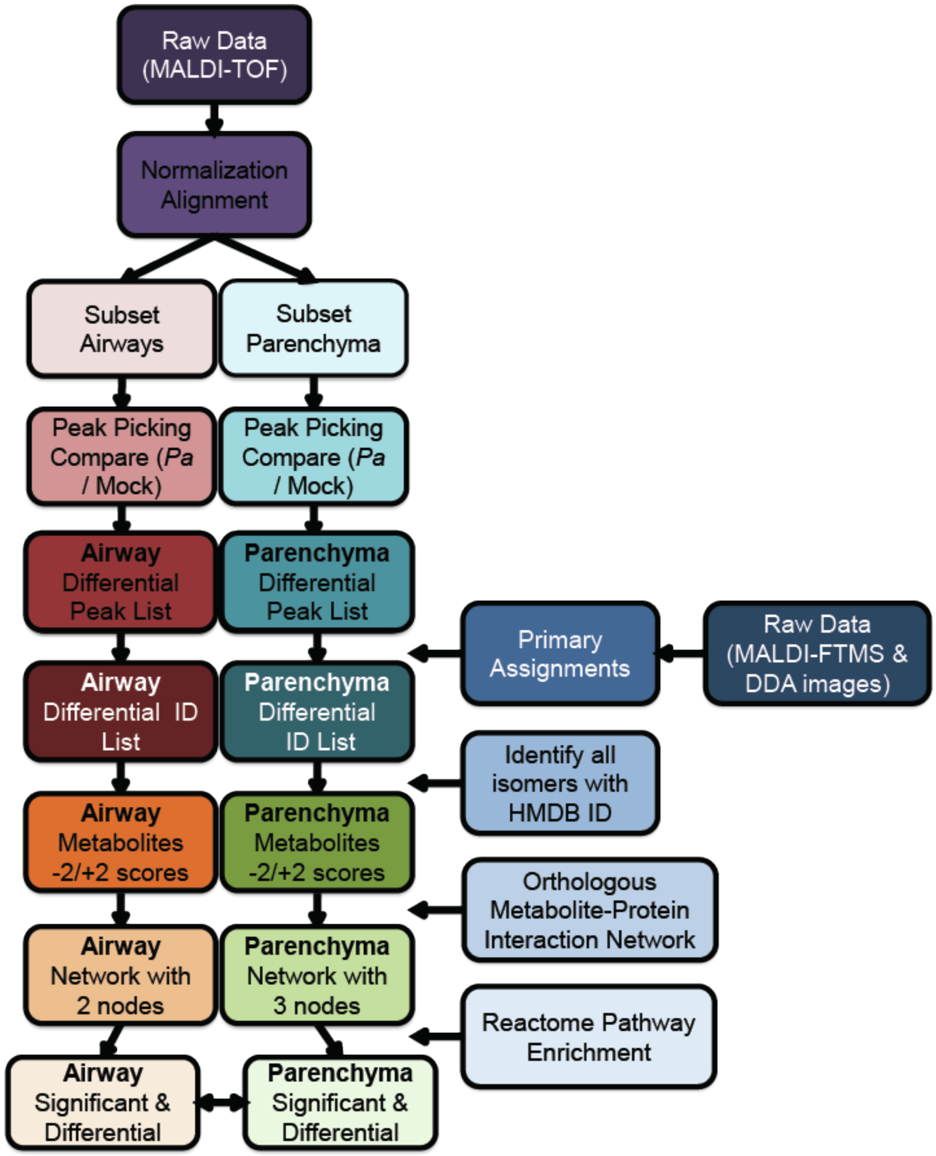

Figure S3: Data processing workflow. Related to Figures 1-2, Table 1.

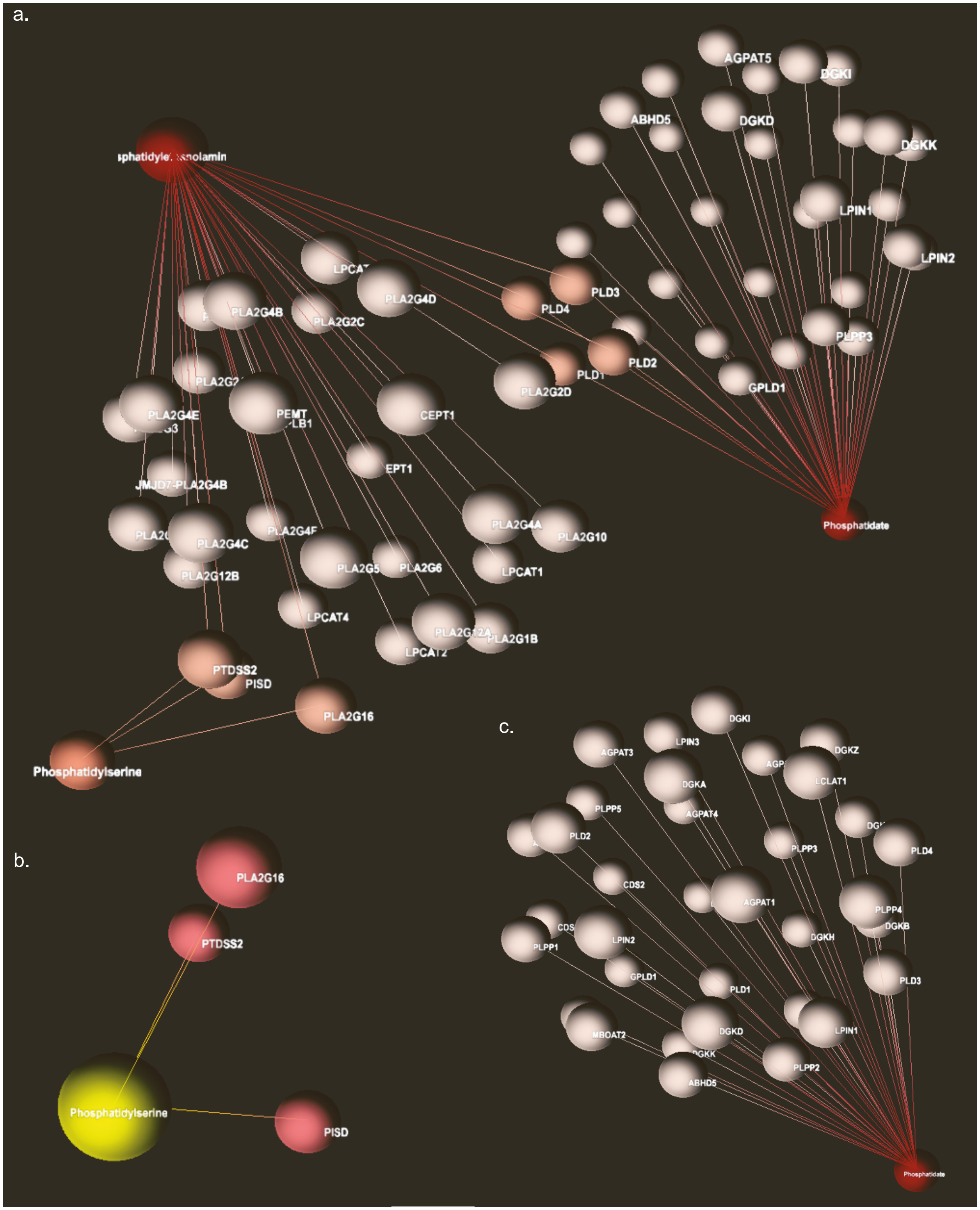

Figure S4: Network construction from seeded metabolite IDs. Related to Figure 2, Table 1. a.) Metabolite-protein interaction network resulting from PCM inputs. b-c.) Two unlinked metabolite-protein interaction networks resulting from AW inputs.

Table S1. Pathway enrichment in AW and PCM. Related to Figure 2, Table 1.

Parenchyma Pathway Enrichment

Pathway name	Entities found (#)	Entities total (#)	Entities ratio	Entities p-value	Entities FDR
Acyl chain remodeling of PC	*23*	*40*	*0.002825857*	*1.11E-16*	*1.22E-15*
Acyl chain remodeling of PE	*21*	*42*	*0.002967149*	*1.11E-16*	*1.22E-15*
Acyl chain remodeling of PG	*15*	*26*	*0.001836807*	*1.11E-16*	*1.22E-15*
Acyl chain remodeling of PS	*17*	*31*	*0.002190039*	*1.11E-16*	*1.22E-15*
Acyl chain remodeling of PI	*14*	*25*	*0.00176616*	*1.11E-16*	*1.22E-15*
Glycerophospholipid biosynthesis	44	232	0.016389968	1.11E-16	1.22E-15
Synthesis of PA	21	65	0.004592017	1.11E-16	1.22E-15
Phospholipid metabolism	44	325	0.022960085	1.11E-16	1.22E-15
Metabolism of lipids	48	1443	0.101942776	1.11E-16	1.22E-15
Metabolism	48	3635	0.256799717	1.67E-15	1.50E-14
Effects of PIP2 hydrolysis	10	40	0.002825857	5.88E-15	4.71E-14
Hydrolysis of LPC	*6*	*18*	*0.001271635*	*3.60E-10*	*2.52E-09*
Synthesis of PE	6	33	0.002331332	1.29E-08	9.06E-08
Synthesis of PG	5	21	0.001483575	5.96E-08	3.57E-07
Platelet activation, signaling and aggregation	11	299	0.021123278	1.37E-07	8.25E-07
Synthesis of PC	6	65	0.004592017	6.73E-07	3.36E-06
G alpha (q) signaling events	10	282	0.019922289	7.50E-07	3.75E-06
Role of phospholipids in phagocytosis	7	129	0.009113387	2.54E-06	1.27E-05
Fc gamma receptor (FCGR) dependent phagocytosis	7	193	0.013634758	3.37E-05	1.35E-04
Acyl chain remodeling of CL	3	18	0.001271635	8.86E-05	3.54E-04
Depolymerization of the Nuclear Lamina	3	23	0.001624868	1.82E-04	7.27E-04
Triglyceride metabolism	4	66	0.004662663	2.70E-04	0.001081992
Triglyceride biosynthesis	3	33	0.002331332	5.20E-04	0.002078028
Hemostasis	11	826	0.058353939	0.00144929	0.004347871
COPI-independent Golgi-to-ER retrograde traffic	*3*	*63*	*0.004450724*	*0.003272943*	*0.009818829*
Nuclear Envelope Breakdown	3	65	0.004592017	0.003570998	0.010712994
Sphingolipid *de novo* biosynthesis	3	79	0.005581067	0.006121946	0.018365838

Airway Pathway Enrichment

Pathway name	#Entities found	#Entities total	Entities ratio	Entities pValue	Entities FDR
Glycerophospholipid biosynthesis	17	129	0.011574697	1.11E-16	2.00E-15
Effects of PIP2 hydrolysis	10	27	0.002422611	1.11E-16	2.00E-15
Phospholipid metabolism	17	212	0.019021983	1.11E-16	2.00E-15
Metabolism of lipids	21	746	0.066935846	2.22E-16	3.11E-15
Synthesis of PA	8	39	0.003499327	4.86E-13	5.84E-12
Synthesis of PG	5	8	7.18E-04	6.08E-11	6.08E-10
G alpha (q) signaling events	10	219	0.019650067	1.00E-09	9.04E-09
Platelet activation, signaling and aggregation	10	262	0.0235083	5.52E-09	4.42E-08
Metabolism	21	2132	0.191296546	1.15E-07	8.03E-07
Role of phospholipids in phagocytosis	6	114	0.010228802	1.44E-06	8.65E-06
Triglyceride metabolism	4	38	0.003409601	6.50E-06	3.90E-05
Synthesis of PE	3	13	0.001166442	1.01E-05	5.05E-05

Triglyceride biosynthesis	3	14	0.001256169	1.26E-05	6.29E-05
Fc gamma receptor (FCGR) dependent phagocytosis	6	175	0.015702109	1.64E-05	6.58E-05
Depolymerization of the Nuclear Lamina	3	16	0.001435621	1.87E-05	7.48E-05
Hemostasis	10	722	0.064782414	5.30E-05	2.12E-04
Synthesis of PC	3	28	0.002512337	9.77E-05	3.91E-04
Sphingolipid de novo biosynthesis	3	44	0.003947959	3.66E-04	0.001099171
Nuclear Envelope Breakdown	3	58	0.005204127	8.14E-04	0.002443138
GPCR downstream signaling	*10*	*1154*	*0.10354419*	*0.002263756*	*0.006791269*
Sphingolipid metabolism	*3*	*90*	*0.00807537*	*0.002841262*	*0.008523786*
Signaling by GPCR	*10*	*1217*	*0.109196949*	*0.003354666*	*0.010063997*
Acyl chain remodeling of PE	2	29	0.002602064	0.003805018	0.011415054
Mitotic Prophase	*3*	*119*	*0.010677434*	*0.006173383*	*0.012346766*
Synthesis of PI	*1*	*5*	*4.49E-04*	*0.015582942*	*0.031165883*
Acyl chain remodeling of CL	1	6	5.38E-04	0.018671099	0.037342197
ChREBP activates metabolic gene expression	*1*	*8*	*7.18E-04*	*0.024819206*	*0.049638411*

*Rows in *italics* are unique to histological region. Data underlying “Differential Pathway Enrichment” histogram (Fig. 2).

Table S2: Tabulated protein list from pathway enrichment for PCM differentially enriched segment and general enzymatic function. Related to Figure 2, Table 1.

Entities	General Function
AGPAT3	acyltransferase
LPCAT1	acyltransferase
LPCAT2	acyltransferase
LPCAT3/MBOAT5	acyltransferase
LPCAT4/MBOAT2	acyltransferase
LPEAT/MBOAT1	acyltransferase
PLA2G10	phospholipase A2
PLA2G12A	phospholipase A2
PLA2G16	phospholipase A2
PLA2G1B	phospholipase A2
PLA2G2A	phospholipase A2
PLA2G2D	phospholipase A2
PLA2G2E	phospholipase A2
PLA2G2F	phospholipase A2
PLA2G3	phospholipase A2
PLA2G4A	phospholipase A2
PLA2G4B	phospholipase A2
PLA2G4C	phospholipase A2
PLA2G4D	phospholipase A2
PLA2G4E	phospholipase A2
PLA2G4F	phospholipase A2
PLA2G5	phospholipase A2
PLA2G6	phospholipase A2
PLB1	phospholipase B1
